# *In vitro* fibrillogenesis of tropocollagen type III in collagen type I affects its relative fibrillar topology and mechanics

**DOI:** 10.1038/s41598-017-01476-y

**Published:** 2017-05-03

**Authors:** Meisam Asgari, Neda Latifi, Hossein K. Heris, Hojatollah Vali, Luc Mongeau

**Affiliations:** 1 Department of Mechanical Engineering, McGill University, 817 Sherbrooke Street West, Montreal, QC H3A 0C3 Canada; 2 Department of Bioengineering, McGill University, 817 Sherbrooke Street West, Montreal, QC H3A 0C3 Canada; 3 Department of Anatomy & Cell Biology, McGill University, 3640 University Street, Montreal, QC H3A 2B2 Canada

## Abstract

Tropocollagen types I and III were simultaneously fibrilized *in vitro*, and the differences between the geometric and mechanical properties of the heterotypic fibrils with different mixing ratios of tropocollagen III to I were investigated. Transmission electron microscopy was used to confirm the simultaneous presence of both tropocollagen types within the heterotypic fibrils. The incorporation of collagen III in I caused the fibrils to be thinner with a shorter D-banding than pure collagen I. Hertzian contact model was used to obtain the elastic moduli from atomic force microscope indentation testing using a force volume analysis. The results indicated that an increase in the percentage of tropocollagen III reduced the mechanical stiffness of the obtained fibrils. The mechanical stiffness of the collagen fibrils was found to be greater at higher loading frequencies. This observation might explain the dominance of collagen III over I in soft distensible organs such as human vocal folds.

## Introduction

The mechanical strength and elasticity of biological tissues are governed by their constituting protein molecules, only a few nanometers in size, which self-assemble to form fibrils with a hierarchical structure. The entanglement of the fibrils is reflected in the overall tissue matrix, which inherits its mechanical properties from the concentration, orientation, and the stiffness of individual protein strands. Collagen is the most abundant protein in the human body. It is the main structural component of various soft tissues such as heart valves, skin, tendon, bone, cornea, lung and the vasculature as well as hard tissues such as bone^1–6^ There are more than 28 types of collagen, among which fibril-forming collagens such as types I and III play a major role in the structural mechanics of human tissues^7–14^ These fibril-forming collagens are distinguished by a repeating banding pattern with a so-called D-periodicity distance (D-banding) of 64–67 nm^1, 12, 15^. Within each collagen fibril, tropocollagen molecules of length ≈300 nm and width ≈1.5 nm are arranged relative to their neighbours by multiples of the D-banding distance.

**Figure 1:**
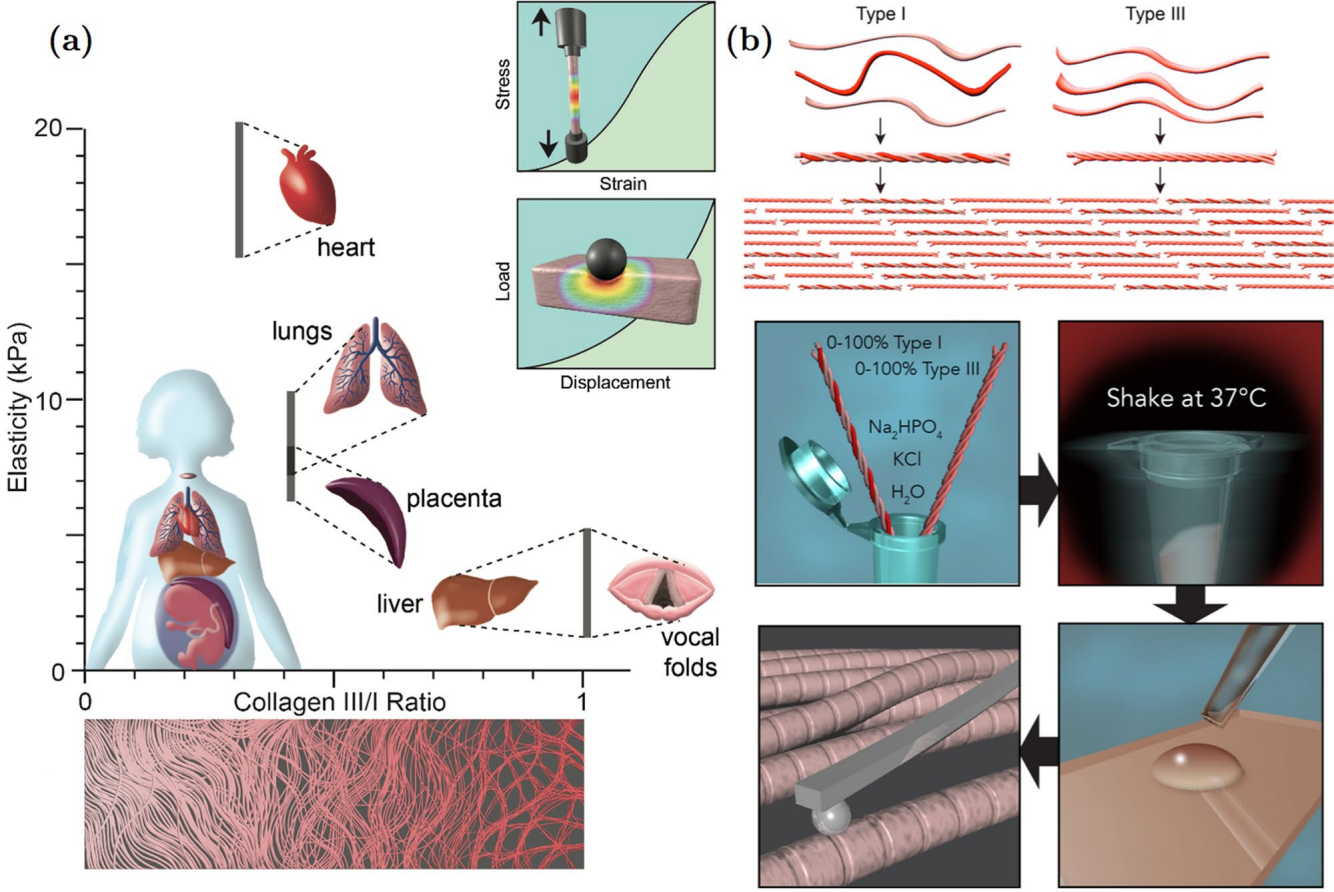
(**a**) Elasticity of various soft tissues in the human body versus the ratio of collagen type III to collagen type I^19, 40, 67–70^; Schematics of conventional tensile and indentation tests, which are widely used for mechanical characterization of soft tissues; (**b**) Tropocollagen types I and III are shown on the top followed by a schematic of their arrangements to form a heterotypic collagen fibril. The bottom part shows the procedure used to fibrilize tropocollagen types I and III *in vitro*, followed by sample preparation for atomic force microscopy imaging and nanoindentation. The resulting fibrils include both of the collagen types I and III.

Each fibrillar collagen molecule is formed by three polypeptide chains (referred to as *α*-chains) with a triple-helical structure. Collagen molecules are categorized as heterotypic, consisting of up to three genetically distinct *α*-chains such as those of collagen type I (Col-I), or homotrimeric, consisting of three identical *α*-chains such as those of collagen type III (Col-III), as shown in Fig. 1 ^1, 10^. Collagen type I, the most widely occurring fibril-forming collagen, is found in the shape of long fibrils in various tissues such as heart, tendon, skin, bone, lung, cornea, and the vasculature^11–13, 16, 17^. Collagen type I molecules are composed of two identical *α*-1 chains, [*α*-1 (I)]_2_, and one distinct *α*-2 chain, *α*-2 (I). Collagen type III is widely present in collagen I-containing tissues with the exception of bone. This fibril-forming collagen is rare in hard tissues and ubiquitous in various elastic tissues such as embryonic skin, lung, and blood vessels, and those found in distensible organs such as vocal folds, bladder, and uterus^18–20^. Also, there is evidence for co-expression of collagen types II and III in human articular cartilage^21–25^. Collagen type III molecules are composed of three identical *α*-1 chains, [*α*-I (III)]_3_^1, 4, 5, 10, 26^.

Although the biochemical composition of Col-III is not radically different from that of Col-I, the presence of Col-III in some differentiated organs or during physiological processes is crucial. Collagen type III is expressed developmentally and during wound healing^18, 27–32^. Previous studies have demonstrated that Col-III might be co-expressed with Col-I to form heterotypic fibers (Fig. 1)^28, 33^. This co-expression may result in the regulation of the dimensions of the fibrils, such as their diameter^27, 34, 35^. Such changes in the geometry and composition of the fibrils may affect their mechanical properties and thus, the functionality of the organ^28, 33^. A certain amount of Col-III is thus reported to be necessary to maintain the normal tension and contraction development within the organ^27, 33^. For example, a shortage of Col-III against Col-I in the bladder changes the fibril size distribution within that organ, which leads to greater compliance and less effective neurotransmitter function^28^. Collagen type III is also reported to modulate scar formation^36–39^. Humans with Col-III mutations have impaired healing, which is likely to be accompanied by excessive scar formation.

It is well established that the viscoelastic properties of the extracellular matrix regulates cellular physiology and the associated tissue hemostasis. Various tissues mechanical properties have been also shown to correlate with the viscoelastic properties of the associated structural collagen fibrils^40^. Atomic Force Microscopy (AFM) has been previously used to study the morphological and viscoelastic properties of Col-I fibrils in various human tissues^41–48^. Taatjes *et al*.^49^ used AFM imaging to investigate the morphological properties of Col-III fibrils.

One may hypothesize that most of the biological and biomechanical functions of organs containing Col-III may have been enabled by nanoscale assemblies with Col-I fibrils. However, to date, little is known about the structural interactions between collagen types I and III that leads to the formation of the heterotypic fibrils found in various soft tissues (Fig. 1)^11, 35, 50, 51^. The purpose of the present study was to investigate the effects of incorporating Col-III in Col-I fibrillogenesis for six different mixing ratios of Col-III:Col-I, which were chosen based on those found in various soft tissues as indicated in Fig. 1a, on the mechanical properties and geometric features of the resulting fibrils. We observed that Col-III has significantly different elastic and dynamic mechanical properties than Col-I. The incorporation of Col-III was shown to soften Col-I fibrils depending on the relative concentration of Col-III to Col-I monomers. Analysis of transmission electron microscopy (TEM) images of Col-I and Col-III revealed that Col-I fibrils are 150–300 nm in diameter, while Col-III fibrils have the diameter of 25–100 nm.

## Results and Discussion

**Figure 2:**
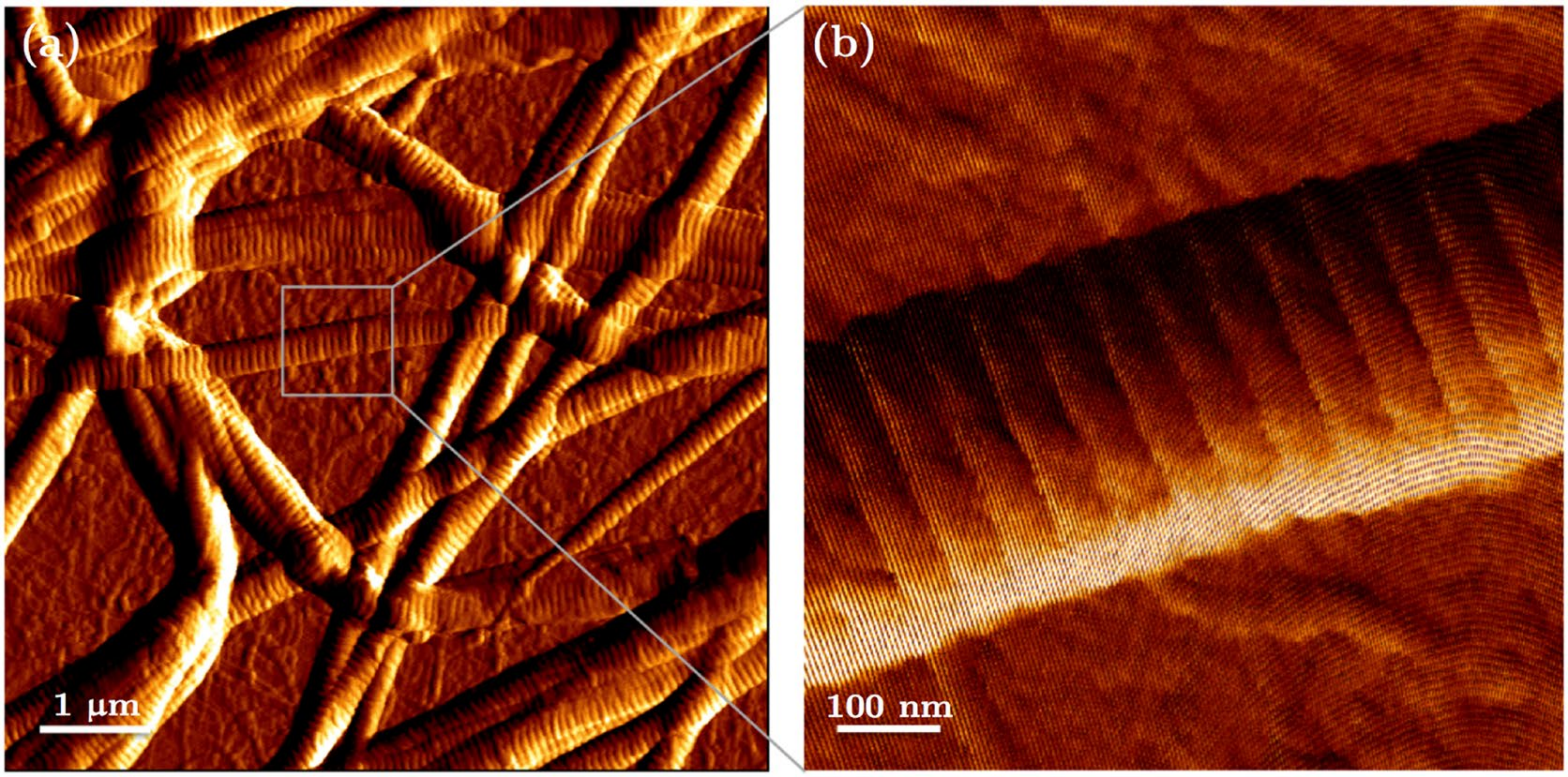
(**a**) Image of a network of collagen type I fibrils obtained with AFM; (**b**) AFM image of one single collagen type I fibril.

**Figure 3:**
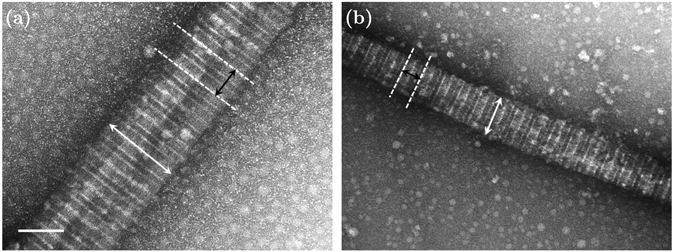
Representative transmission electron microscopic (TEM) images of collagen fibrils (magnification: 50000). (**a**) 100% collagen type I in the solution; (**b**) Col-III:Col-I of 50:50; The average topology of the fibrils such as their diameter and D-banding slightly changes when adding tropocollagen type III to the solution. The scale bar represents 100 nm.

**Figure 4:**
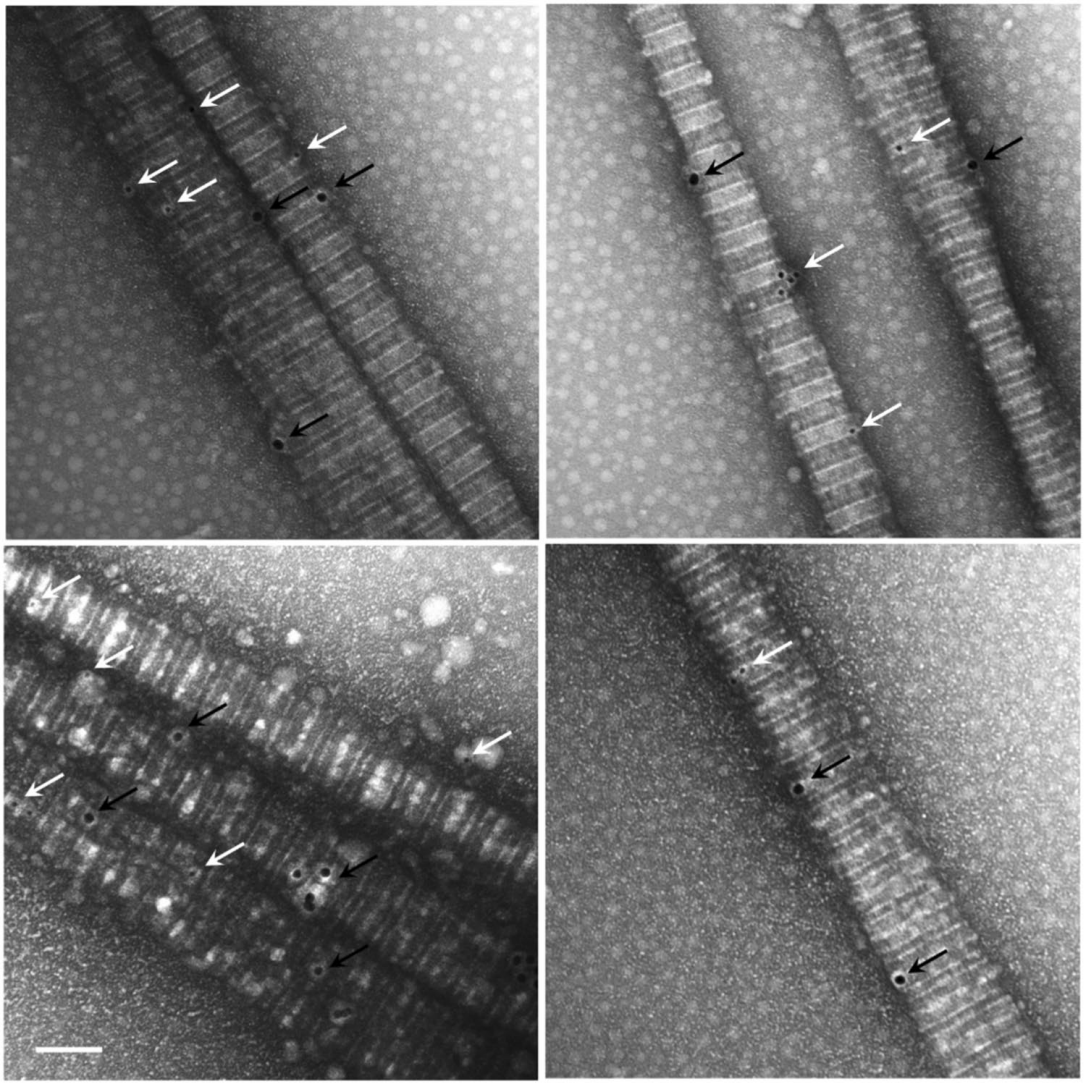
Representative transmission electron micrograph of fibrilized collagen with a Col-III to Col-I ratio of 50:50%. Col-I and Col-III were stained using gold nanoparticles of 18 nm and 12 nm in diameter, respectively. The larger dots represent the particles attached to tropocollagen type I, as pointed using black arrows, whereas the smaller ones are attached to tropocollagen type III, as pointed using white arrows. The scale bar represents 100 nm.

Figures 2 and 3 show representative AFM and TEM images of the collagen fibrils, respectively. Fibril diameters are indicated in Fig. 3. We observed a slight decrease in both diameter and D-banding with an increase in the Col-III:Col-I ratios. Figure 4 shows representative TEM images of the immuno-stained collagen fibrils obtained with a primary Col-III:Col-I mixing ratio of 50:50. The larger dots represent the gold nano-particles attached to Col-I, as pointed using black arrows, whereas the smaller dots represent those attached to Col-III, as pointed using white arrows. We observed the co-existence of collagen types I and III in the resulted fibrils from the primary mixture of both these tropocollagen types. Our results confirmed the formation of heterotypic fibrils of Col-I and Col-III, which were then studied for their geometrical and mechanical features.

**Figure 5:**
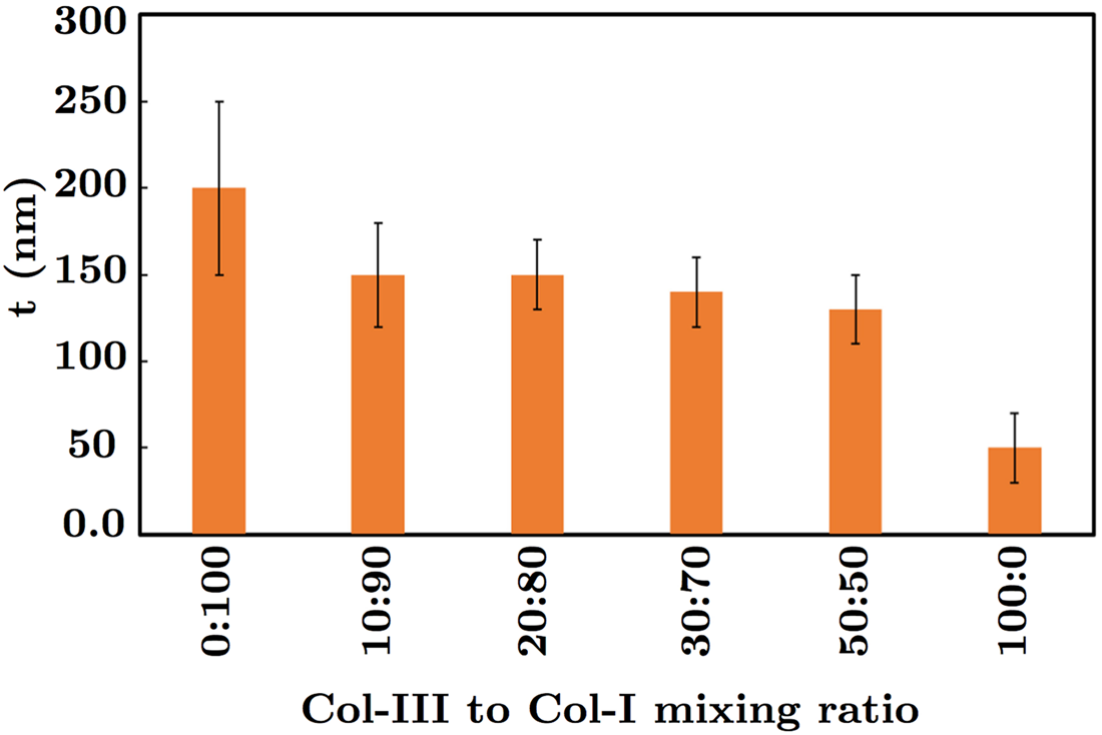
Average diameter t of collagen fibrils with different ratios of the tropocollagen molecules type III to I (Col-III:Col-I).

**Figure 6:**
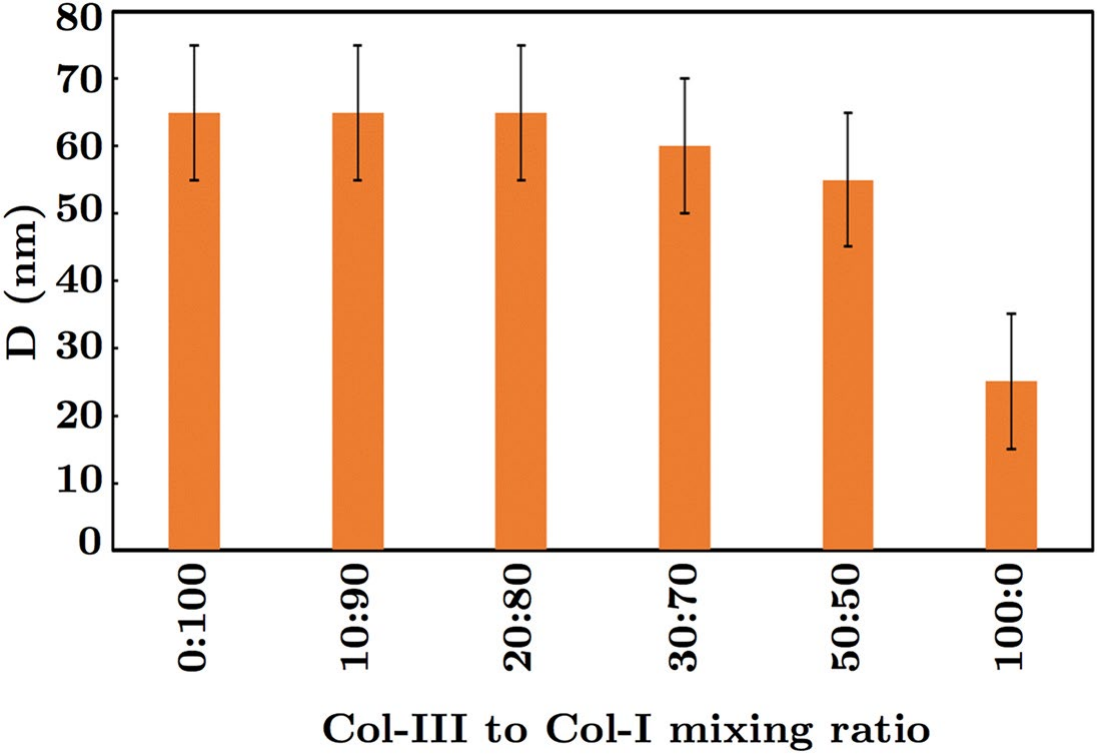
The graph of the measured axial periodicity (i.e., D-banding) D of collagen fibrils for different ratios of tropocollagen types I and III.

The average diameter of collagen III fibrils (Col-III:Col-I of 100:0) was found to be smaller than that of collagen type I fibrils (Col-III:Col-I of 0:100), as shown in Fig. 5. The differences between the diameter of Col-I and Col-III fibrils were statistically significant (P < 0.05). The fibril diameter was decreased by increasing the primary Col-III:Col-I mixing ratio for the heterotypic fibrils. However, no statistically significant differences were observed between the diameters of Col-I fibrils and those of heterotypic fibrils obtained from a primary Col-III:Col-I mixing ratio of less than 50:50. Previous studies on the collagen content of mice tail, skin, or bladder have shown larger diameters for the heterotypic collagen fibrils of mice with a Col-III deficiency compared to those of the controls^28, 35^. Romanic *et al*. also reported thinner fibrils obtained from the co-polymerization of collagen types I and III^34^. The slight decrease in heterotypic fibril diameter following the tropocollagen type III addition may be explained based on a concentric model, known as the Hulmes’s model, of molecular packing in collagen fibrils^9, 52^. Hulmes’s model was derived based on energy minimization and according to experimental data from X-ray diffraction and electron microscopy tests on tendon collagen fibrils. The fibril surface was assumed to be coated by the molecular ends, which helps explain the fibril growth. Collagen types I and III solutions used in the present study are enzymatically extracted from the ECM secreted by neo-natal fibroblast cells. The tropocollagen molecules are therefore cleaved at the telopeptides, and both the N-terminal and C-terminal propeptides are assumed to be removed. But, N-terminal processing for the collagen type III molecules is reported to be relatively slow^9^. Delayed N-terminal processing may result in the accumulation of a partially processed form of collagen molecule, which lacks the C-terminal propeptides but retains the N-terminal propeptides. The C-terminal propeptides are controlling the solubility of the collagen molecules and prevent collagen fibril formation. However, the N-terminal propeptides do not prevent fibril formation. They presumably affect the fibril shape and diameter. Persistence of the N-propeptide may prevent their incorporation in the center of the fibril. Therefore, all N-termini are forced to the fibril surface, which prevents further accretion and limits fibril diameter^53^. This may explain the mechanism by which the heterotypic fibrils of collagen types I and III have smaller diameters compared to that of Col-I fibrils^27, 28, 34, 52^. We obtained D-banding values of ≈65 ± 5 nm and 25 ± 10 nm for Col-I and Col-III fibrils, respectively, as shown in Fig. 6. The D-banding was slightly decreased for collagen fibrils with Col-III:Col-I ratios of less than 50:50 compared to that of Col-I fibrils. The differences between the measured D-banding distances of collagen fibrils with Col-III:Col-I ratios of less than 50:50 and Col-I fibrils were statistically insignificant (P > 0.05). The average D-banding distance, however, decreased following the addition of tropocollagen type III to the primary mixture. The differences between the D-banding of Col-I and Col-III fibrils were statistically significant (P < 0.05). The composition of the heterotypic fibrils therefore affects their D-banding distance. Our results may help explain the differences between the D-banding distance of different soft tissues reported in the literature^54^. The average fibril diameter and D-banding decreased with an increase in Col-III:Col-I mixing ratio, as shown in Figs 5 and 6, respectively.

**Figure 7:**
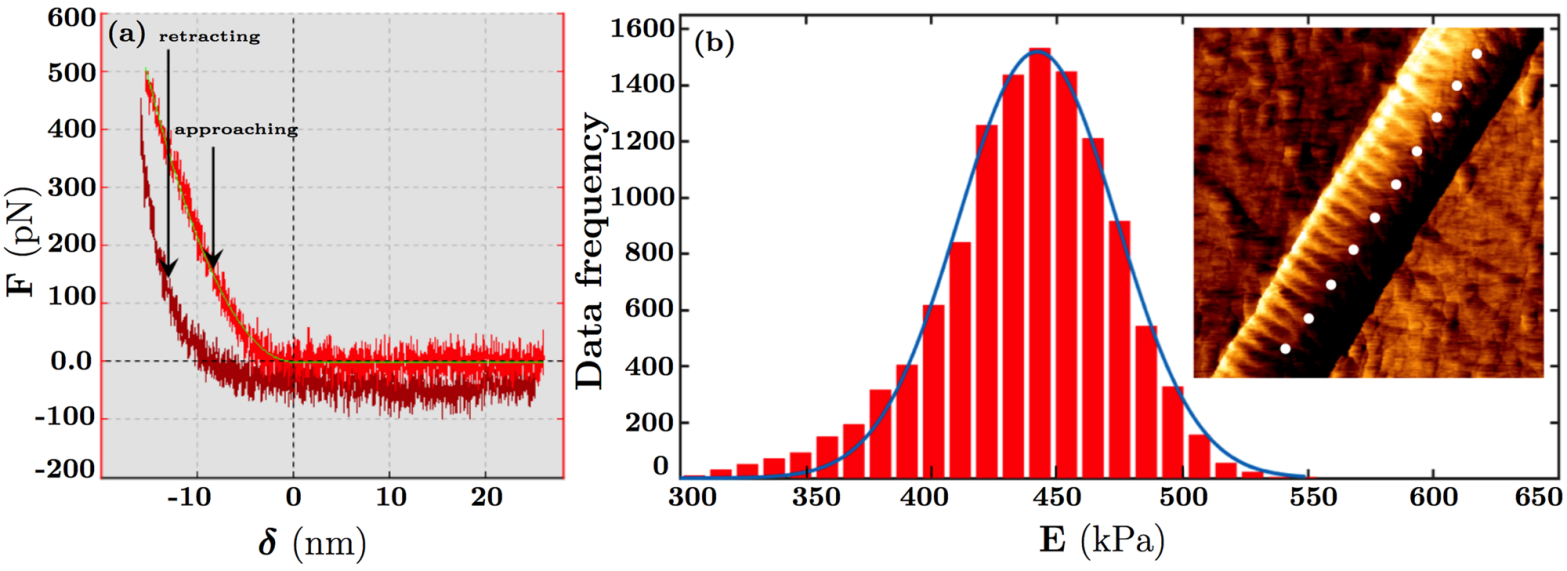
Representative atomic force microscopy results. (**a**) Schematic of the vertical deflection extend, F, in terms of the tip-sample separation, δ; (**b**) Plot of the Gaussian distribution versus the elastic modulus, E, for fibrils with the ratio of tropocollagen I/tropocollagen III = 9:1 in Phosphate Buffered Saline (PBS) solution (The red diagram denotes the histogram of the data, and the blue curve represents the fitted Gaussian curve). A representative force map including some indentation points on a collagen fibril is displayed.

**Figure 8:**
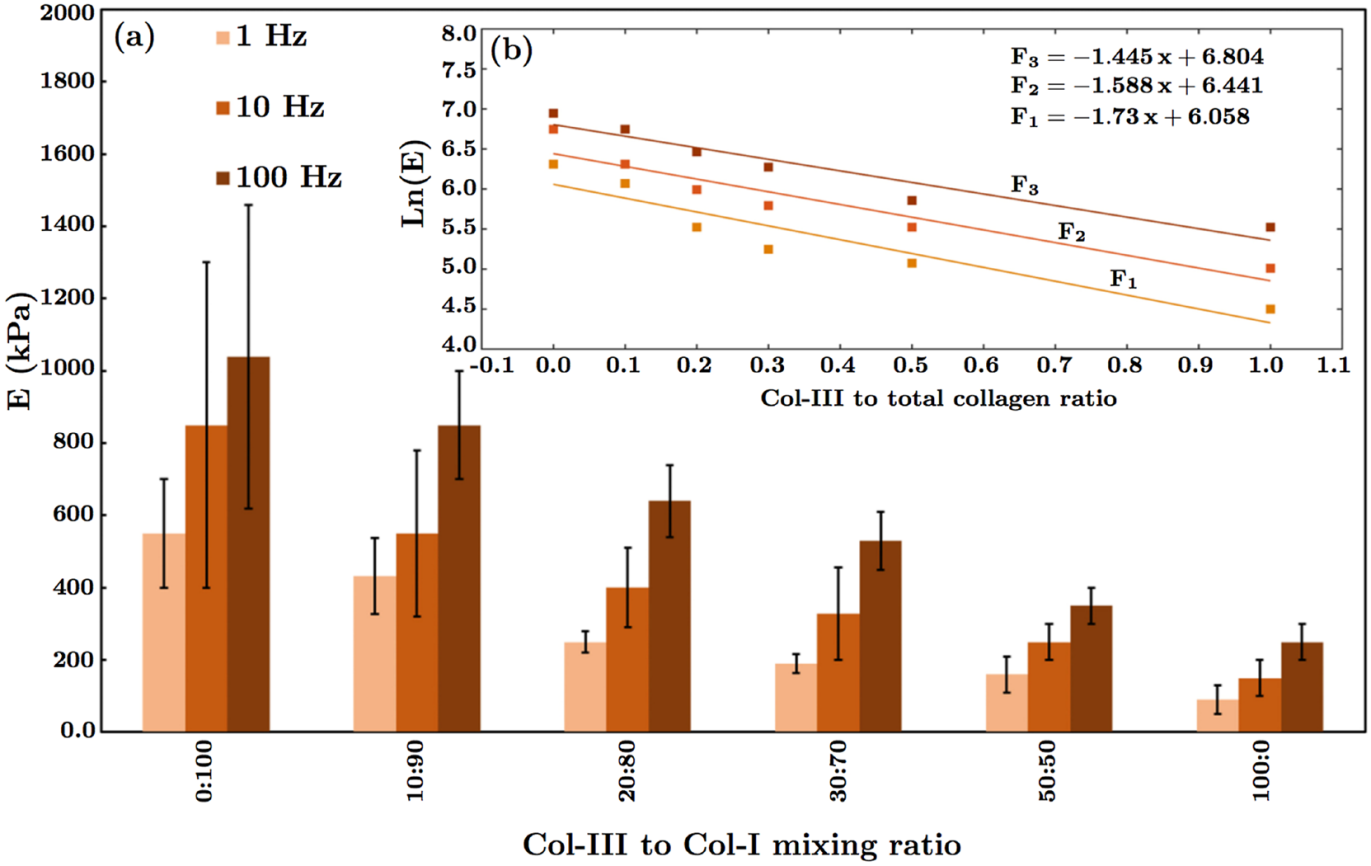
Measured dynamic elastic moduli of collagen fibrils for different loading rates (1–100 Hz). (**a**) Elastic moduli versus Col-III:Col-I mixing ratio expressed as the mean ± standard error. The differences were found to be statistically significant (p < 0.05); (**b**) The semi-logarithmic diagrams of the average elastic moduli versus Col-III:Col-I mixing ratios and the fitted curves. *F*1, *F*2, and *F*3 denote the logarithmic function of the elastic modulus *E* at 1, 10, and 100 Hz, respectively. The elastic moduli obtained at 100 Hz were found to be greater than the ones obtained at 10 Hz. Similarly, the moduli obtained at 10 Hz were greater than the ones obtained at 1 Hz.

Figure 7 shows representative atomic force microscopy results. A schematic of the vertical deflection extend, *F*, in terms of the tip-sample separation, *δ*, is shown. A representative plot of the Gaussian distribution versus the elastic modulus, E, for a single heterotypic collagen fibril with a Col-III:Col-I ratio of 10:90 is also shown. The red diagram denotes the histogram of the experimental data, and the blue curve represents the fitted Gaussian curve. The measured elastic moduli for different Col-III:Col-I mixing ratios for three different loading rates are shown in Fig. 8. The value of the elastic modulus was obtained using the Hertzian contact model, considering the Poisson’s ratio to be ≈0.5. The values were calculated from the force curves taken along the central spine of the fibril to avoid geometric effects. Isolated fibrils of height larger than 80 nm and less than 150 nm were selected for our analysis, as no change in elastic modulus was detected in this range. The cantilever spring constant was determined, and the cantilever was calibrated accordingly.

The elastic modulus for Col-I fibrils immersed in PBS ranged from 400 kPa to 1 MPa. The Gaussian regression functions reached a maximum at 550 ± 150 kPa. Indentation measurements on the same sample at higher indentation frequencies led to a larger stiffness and a narrower distribution ranging from 600 kPa to 1.5 MPa with a peak at 1050 ± 400 kPa. Our results indicate a good correspondence with most of the previously obtained values of the elastic modulus for Col-I fibrils in the hydrated state^55–58^. As reported in previous studies, the mechanical properties of collagen fibrils depend upon the presence of fluid on the sample, but geometric features such as D-banding do not. Thus, the value of the elastic modulus for the dehydrated or mineralized samples of collagen type I fibrils are expected to be about three orders of magnitude greater than our obtained results, which are for the samples immersed in PBS^13, 59–63^.

It was found that the indentation frequency directly affects the measured mechanical stiffness. This describes the apparent elasticity of the collagen according to the loading rate in a quantitative way. For the same force applied, the indentation depth decreases as the loading rate increases from 1 to 100 Hz. This observation suggests that collagen appears to be stiffer at a higher loading rate. As shown in Fig. 8b, we found an exponential decrease in elastic modulus versus Col-III:Col-I ratio. The increase in elastic modulus with frequency was postulated to be associated with the viscoelasticity of the fibrils. Collagen fibrils are physically cross-linked dense biopolymer hydrogel networks. Previous studies have shown that dehydration results in a large increase in their elastic modulus. At low frequencies, the cross-linked network has enough time to reach an equilibrium under load, as water has sufficient time to move out of the dense structure, and collagen molecules rearrange accordingly. It is widely believed that the modulus of cross-linked biopolymer networks reaches a plateau at low frequencies (f < 1 Hz). However, such an equilibrium state may not be reached at high frequencies, when collagen molecules may not have enough time to rearrange, and dense fibrillar structures exhibit a higher resistance to water movement leading to a stress-stiffening behaviour. Detailed studies of viscoelastic properties of collagen fibrils and associated mechanical models should be subject of future research.

Elastic modulus describes the resistance of a linearly isotropic elastic material subjected to a given axial stress, against deformation. For instance, stiff materials in tendons transmit forces and resist deformation. In particular, when a material is subjected to bending or buckling, stiffness is crucial for transmitting forces. For this reason, hard tissues like bone are reinforced with highly stiff mineral particles. Although assumptions such as homogeneity, isotropy, and linear elasticity cannot be completely guaranteed when applying the Hertz’s model to analyze the AFM indentation data from *soft materials*, this model is still widely accepted to approximate the elastic modulus when small deformation assumption is satisfied^41, 64^.

It was found that the incorporation of tropocollagen type III in fibrillar collagen type I has a significant effect on the resulting fibrillar topology and its mechanical stiffness. According to previous studies, the lack of collagen type III disturbs fibrillogenesis, and may result in functional failure of the organs. In physiological conditions, collagen type III is an essential fibrillar component of tissues such as aorta. It is also a significant regulatory element in collagen type I fibrillogenesis. Our results provide a quantitative assessment of the previously-reported qualitative finding that collagen type III regulates the topology of collagen type I fibrils^35^. This phenomenon serves as a mechanism to meet the physiological conditions and requirements of different tissues or those of a particular tissue at different developmental stages. Dynamically sollicited organs such as vocal fold in which the oscillatory motion often takes place, include more collagen type III than type I. An air-flow induced vocal fold bioreactor was recently developed^65^, which mimics the dynamic biomechanical micro-environment of the human vocal folds. As part of our future direction, we intend to use the bioreactor to stimulate cell-seeded scaffolds with a fibrillar structure composed of collagen fibrils with different mixing ratio of tropocollagen types I and III to improve our current understanding of the influence of Col-III in human tissues composed of heterotypic collagen fibrils.

## Materials and Methods

### Collagen fibrillization

Tropocollagen monomers type I (3 mg/mL in 0.01 N HCl, pH 2) and type III (1 mg/mL in 0.01 N HCl, pH 2) from human placenta in acidic solutions were purchased from Advanced BioMatrix Inc., Carlsbad, CA. To fibrillize the collagen monomers, a recently published protocol by Loo *et al*.^66^ was applied. First, 20 L of 200 mM disodium hydrophosphate (Na_2_ HPO_4_, pH 7), 6 L of sterile deionized water (ddH_2_ O), and 10 L of 400 mM potassium chloride (KCl) were combined and mixed in an eppendorf centrifuge vial. Then, 4 L of tropocollagen solution was added to obtain the Col-III:Col-I ratios of 0:100, 5:95, 10:90, 20:80, 30:70, 50:50 and 100:0. The solution was properly mixed at room temperature. The vial was then placed inside a commercial cell culture incubator at 37 °C for 6 hours. A total amount of 4 L of collagen monomers with varying tropocollagen types I and III volumes were used.

### Atomic Force Microscopy

A JPK Atomic Force Microscope (JPK Nano-wizard@3 Bio-Science, Berlin, Germany) was used for imaging and force spectroscopy. To prepare the collagen samples for Atomic Force Microscopy (AFM), 20 L of the fibrilized collagen was placed on a microscope slide. After 15 minutes, the sample was gently washed with deionized water to eliminate the salt residues. Collagen fibrils are highly attracted to other surfaces, due to their electrostatically charged amino acids. Hence, the fibrils adhered strongly to the slide. Appropriately-positioned fibrils, which did not overlap, were identified and located to perform force measurements. Prior to the indentation force measurements, 200 L of phosphate buffer solution (PBS 1X) was added to the sample to hydrate the fibrils for 30 minutes. All the measurements were performed in PBS 1X. Using the QI imaging mode of the JPK AFM, a force map was created within the area of 10 m^2^ on the sample containing various fibrils. Only the points located on the selected fibrils were indented, for consistency. A Nanotools CONTR B50 cantilever with a 50 nm ± 10% defined spherical tip (Nanotools USA LLC, Henderson, NV) was used for indentation. The indentation frequency was 1, 10, and 100 Hz. The indentation was repeated at the same location for consistency as well to ensure that the fibrils were not permanently deformed. The indentation depth depends on the applied load, as well as the stiffness of the tip and that of the sample. The elastic modulus of the fibril was estimated from the approaching force-indentation depth curve. According to the Hertzian contact model, the elastic modulus, *E*, of the sample is given in terms of the applied indentation force, *F*, by $E=3F\mathrm{(1}-{\nu }^{2}\mathrm{)/4}\sqrt{r{\delta }^{3}}$, where *ν* is the Poisson’s ratio of the sample (selected to be 0.5 assuming incompressibility), *δ* represents the indentation depth, and *r* denotes the effective contact radius, expressed as $\mathrm{1/}r=\mathrm{1/}{r}_{1}+\mathrm{1/}{r}_{2}$, in terms of *r*_1_ and *r*_2_, the radii of the two contacting solids, i.e., the used AFM probe (in our case 50 nm) and the collagen fibril. Indentation tests were performed at different frequencies to measure the dynamic elastic modulus. Hydrodynamic drag forces at both low and high frequencies were negligible (1–5 pN) and were ignored. The deflection sensitivity of the piezo module was established by probing the surface of the glass substrate. A thermal tuning method was used to calibrate the stiffness of the cantilevers. Sharp AFM probes (MSNL10, Bruker) with tip radii of 2–12 nm were used for high resolution imaging of collagen fibrils. AFM data analysis was performed with the native JPK data processing software. Statistical significance was determined by a paired student’s t-test, when applicable. Differences were considered significant at p < 0.05.

### Immuno-Electron Microscopy

Immuno-electron microscopy was performed to double-stain samples for Col-I and Col-III in order to investigate the simultaneous presence of both collagen subtypes in single fibrils in the solutions. The solution was first diluted five times using distilled deionized water. One drop of 5 L of the obtained solution was placed on a glow discharged carbon grid, i.e. a coated 200 Mesh copper electron microscopy grids (supplied by Electron Microscopy Sciences, EMS, Hatfield, PA). After two minutes, the excess solution was removed using a filter paper at the periphery of the grid, and the grid was placed facing up for a duration of 30 minutes. It was then blocked using BCO (2% BSA- 2% Casein and 0.5% Ovalbumin) solution for 5 minutes. The grid was subsequently incubated with a mixture of two primary antibodies, i.e., rabbit monoclonal anti collagen I (ab138492) and mouse monoclonal anti collagen III (ab6310, Abcam Inc., Toronto, ON), with a mixing ratio of 1:1 for 2 hours. It was then washed using 6 drops of filtered Dulbecco’s phosphate buffered saline (DPBS 1X, Life Technologies Inc., Burlington, ON) for 15 minutes. The grid was then blocked again with BCO solution for 5 minutes. The sample was subsequently incubated on one drop of a mixture of two secondary antibodies in BCO (i.e., goat anti rabbit 18 nm gold particles (1:10) and goat anti mouse 12 nm gold particle (1:10) for 1 hour. It was then washed on 6 drops of DPBS 1X for 15 minutes followed by 6 drops of ddH_2_ O for 10 minutes. The grid was then fixed on one drop of 1% Glutaraldehyde in ddH_2_ O for two minutes. It was subsequently washed with 6 drops of ddH_2_ O for 15 minutes. The grid was then dried at an angle on a filter paper, and incubated with counter-stain, 2% Uranyl Acetate, for 30 minutes. The grid was finally dried at an angle on a filter paper, and placed facing up on a filter paper for 30 minutes. A transmission Electron Microscope (TEM, Tecnai 12 microscope (FEI electron optics) equipped with a tungsten filament at 120 kV and an AMT XR80C CCD Camera System) with a magnification of 50000 was used to image the stained samples. The obtained TEM images were analyzed using ImageJ software to measure the fibril diameter and D-banding. For each Col-III:Col-I ratio, 3 × 5 images were analyzed (Three samples were prepared in separate vials, and 5 images of each sample were obtained for further analysis). About 15–20 fibrils were therefore analyzed and the obtained data was averaged. For each fibril, diameter and D-banding were measured at 10 different locations. The pixel size was 0.35461 nm. Regarding image analysis using ImageJ, we used the same brightness/contrast settings for all the images. It was adjusted in ImageJ prior to the measurements. The same examiner did all the analysis in one session to reduce the user related errors. Another examiner subsequently performed several measurements for each study group independently. The obtained diameters and D-bandings were compared with those found by the main examiner. We did not find any substantial differences between these two data sets. It is standard to repeat the image analysis to get data from two (or more) “blind” observers in order to reduce the error associated with variability due to subjective bias.
